# Hemovigilance in a Brazilian Amazon blood center: A temporal analysis of 5-year consumption patterns

**DOI:** 10.1016/j.htct.2026.106460

**Published:** 2026-05-13

**Authors:** Cristie Hellen Alves Cascaes, Thayenne Ribeiro Alves, Jônatas Alencar Castro Campelo, Tatiane Amabile de Lima, Joseir Saturnino Cristino, Alexander Leonardo Silva-Junior, Evilázio Cunha Cardoso

**Affiliations:** a Universidade Estacio de Sá - Campus Amazonas, Manaus, Amazonas, Brazil; b Universidade Nilton Lins, Manaus, Amazonas, Brazil; c Faculdade de Ciências Médicas, Universidade Estadual de Campinas (UNICAMP), Campinas, São Paulo, Brazil; d Fundação Hospitalar de Hematologia e Hemoterapia do Amazonas (HEMOAM), Manaus, Amazonas, Brazil; e Hemocentro da UNICAMP, Campinas, São Paulo, Brazil; f Universidade Federal do Amazonas (UFAM), Manaus, Amazonas, Brazil

**Keywords:** Blood components, Hemotherapy, Blood treatment, Acute treatment, Brazilian amazon

## Abstract

**Background:**

The transfusion of blood components remains a cornerstone in the management of hematological and onco-hematological diseases. Effective blood bank management, combined with the development of predictive models for component utilization, is essential to anticipate local demands, optimize resource allocation, and support the formulation of evidence-based public health strategies.

**Aims:**

To analyze temporal trends in the consumption of blood components at a reference center in the Brazilian Amazon over a five-year period (2019–2023) and to develop predictive models to forecast future demand.

**Methods:**

Transfusion records from the Outpatient and Inpatient Departments were retrospectively collected and categorized by component type. Time-series and regression analyses were applied to assess consumption dynamics. Machine learning models using Auto Regressive Integrated Moving Average and Random Forest algorithms were employed to predict future blood demand.

**Results:**

Overall transfusion demand decreased during the study period; however, irradiated platelet concentrates, cryoprecipitate, and plasma by apheresis showed significant increases, particularly in the Inpatient Unit. These components are predominantly indicated for immunosuppressed patients, such as those undergoing chemotherapy or bone marrow transplantation.

**Conclusion:**

The rising demand for irradiated components reflects the clinical complexity of patients treated in a regional hemotherapy referral center. The findings of this study highlight the importance of forecasting tools in supporting transfusion safety, optimizing blood bank management, and guiding public health strategies in the Amazon.

## Introduction

Transfusion therapy has been used since before the 1900s as a palliative treatment for various medical conditions; however, the majority of transfusion demands are associated with hematological malignancies [1, 2, 3]. While numerous studies have focused on patient-specific responses to blood components, there remains a significant gap in understanding the transfusion profiles and operational dynamics of blood banks. In Brazil, hemotherapy is based on a voluntary, non-paid model as stated by National Politics of Blood and Products [4]. Data regarding blood consumption varies by Brazilian region, with the highest demand in the southeastern and southern regions [5]. The state of Amazonas is the largest by territory, yet it relies on a single blood bank located in the capital, which is responsible for supplying blood to all 39 healthcare units in the capital city, as well as to 54 municipalities across the state and several neighboring regions.

Data from 2021 indicates that over 23,000 liters of blood were distributed in the state of Amazonas, with an average of 235 transfusions performed daily. Of these, approximately 96% took place in the capital, while only 4% occurred in other municipalities across the state [6]. These Fig. underscore the critical importance of blood donation and the need for a deeper understanding of hospital transfusion profiles and patient demand for blood components.

Several guidelines have been established to provide recommendations for the clinical use of blood, with a particular emphasis on the components. Following donation, whole blood is centrifuged and separated into individual components, allowing a single donation to yield red blood cells (RBCs), platelet concentrate (PC), fresh frozen plasma (FFP), and cryoprecipitate (Cryo). These components can also be obtained through apheresis, a technique that yields higher concentrations of specific components but involves greater operational costs. Each of these blood products has distinct therapeutic purposes and plays a critical role in patient management across a variety of clinical settings [7].

Hemovigilance refers to a set of procedures that encompass the entire blood cycle, from donor recruitment to post-transfusion patient follow-up. It is primarily aimed at monitoring and preventing transfusion-related adverse events. This process is essential for maintaining quality standards, ensuring the safety of therapeutic procedures, identifying adverse reactions, and guiding system improvements [8]. As part of hemovigilance, understanding the patterns of blood component usage over time is crucial for planning future donation campaigns and optimizing resource allocation. Despite its importance, few studies have addressed this approach, even though it is well established that blood donation rates fluctuate throughout the year. Managing large datasets and extracting meaningful insights remain significant challenges for blood bank managers, further emphasizing the need for data-informed strategies in both blood donation and usage [9, 10, 11, 12].

This study presents an analysis of hemovigilance data collected over a five-year period (2019–2023) from the regional blood center in the state of Amazonas, Brazil.

## Materials and methods

### Data availability

This is a descriptive and retrospective study based on data concerning blood component requests and transfusion records from the Fundação Hospitalar de Hematologia e Hemoterapia do Amazonas (HEMOAM), located in Manaus, the capital of the state of Amazonas, Brazil. HEMOAM is the only specialized public hospital in the largest state of Brazil and serves most patients with blood disorders, including primarily onco-hematological conditions (such as leukemia, Hodgkin’s and non-Hodgkin lymphomas, and multiple myeloma), as well as sickle cell disease, thalassemia, and coagulation disorders.

Information regarding blood transfusions is recorded daily in the national Hemovigilance software (Hemosys) by authorized hospital staff. This tool manages all transfusion activities within the hospital, and all the data used for analysis was extracted from this software platform. Daily transfusion records from both outpatient (Transfusion) and inpatient (Hospitalization) Departments were collected for the period from January 2019 to December 2023. The Inpatient Department primarily manages patients with acute and chronic leukemia, aggressive lymphoma, severe coagulopathies, and hematopoietic stem cell transplant recipients, all of whom present high transfusion requirements. The Outpatient Department, on the other hand, treats patients with chronic blood disorders, such as sickle cell disease and patients receiving maintenance chemotherapy. Blood components were categorized based on clinical indication and medical specialty, as outlined in Table 1.

**Table 1:** Description of types of blood components used and their management prior to transfusion.

Blood component	Type of component	Description
RBCs	RBCs	Whole blood-derived from blood donations
	Irradiated	Treated with ionizing radiation
	Washed	Washed with isotonic saline
	Filtered	Leukoreduced
	Phenotyped	Phenotyped for ten antigens
PC	PC	Platelets recovered from whole blood donations
	Irradiated	Treated with ionizing radiation
	PCa	Obtained from a single donor via automated apheresis
	Irradiated PCa	Treated with ionizing radiation
FFP		Plasma rich in coagulation factors and plasmatic protein, obtained after double centrifugation and removal of RBCs and PC
Cryo		Precipitated portion of FFP rich in Fibrinogen and Factor VIII

RBC: Red Blood Cell; PC: Platelet Concentrate; PCa: Platelet Concentrates by apheresis; FFP: Fresh Frozen Plasma; Cryo: Cryoprecipitate.

Note: Components requiring multiple interventions (e.g., washed and filtered RBCs) are recorded as separate sub-categories. This classification identifies the specific transfusion requirements of different clinical units and accounts for the cumulative processing steps performed prior to administration.

### Data analysis of crude data

A database was constructed using Microsoft Excel 2010 and analyzed in RStudio. The total number of transfusions was aggregated by day, month, and year over the entire study period. Additionally, the data was stratified by blood component type and by the requesting hospital department. For data visualization, Local Polynomial Regression (LOESS) with standard error bands was applied, and variation curves were generated using appropriate R packages.

### Poisson regression models

Poisson regression models were employed to analyze the association between transfusion counts and hospital departments and to evaluate daily transfusion rates across different hospital departments over the study period. In the first step, the number of transfusions was treated as the dependent variable that included departments as covariates, estimating the log-relative rate of variation over time. Subsequently, the effect of department-specific dynamics was examined by adjusting for the day of transfusion as a covariate. All analyses were conducted using R software, with a 95% confidence interval. Blood components that showed a positive log-relative rate were selected for subsequent forecasting analyses.

### Auto regressive integrated moving average (ARIMA) modeling

To explore time trends and forecast future transfusion demands, ARIMA models were applied to the components identified as increasing in use by Poisson regression. Each ARIMA model was fit separately by component using monthly data from 2019 to 2022, while data from 2023 were used for validation. Optimal model parameters (p, d, q) were selected based on the Akaike Information Criterion (AIC), Bayesian Information Criterion (BIC), and diagnostic plots of the autocorrelation and partial autocorrelation functions. Model performance was assessed using the root mean square error (RMSE) and the Box-Ljung test to evaluate the independence of residuals.

### Random forest

To complement the statistical models and evaluate the predictive performance of machine learning techniques, a Random Forest model was applied to predict transfusion volumes for the selected blood components. The dataset was randomly split, with 80% used for training and the remaining 20% for validation. The model was implemented using default parameters, and variable importance was considered to interpret the most influential features in the prediction of transfusion demand.

## Results

### Blood component use over the years: descriptive dynamics of crude values

**Fig. 1:**
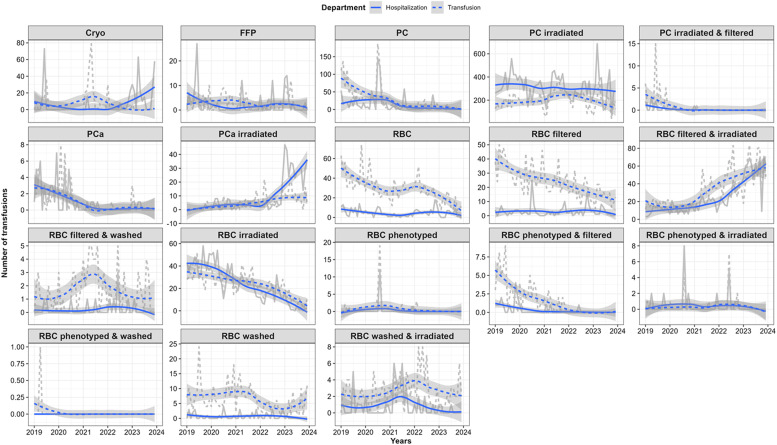
Descriptive data regarding blood usage. The dynamics in both hospital departments were reported daily from 2019–2023. Crude data of each blood component/day are represented by the grey lines, and Locally Estimated Scatterplot Smoothing (LOESS) by blue lines. Departments are categorized by line style as Inpatient (continuous) and Outpatient (dashed). Cryo: Cryoprecipitate; FFP: Fresh Frozen Plasma; PC: Platelet Concentrate; PCa: Platelet Concentrate by apheresis; RBC: Red Blood Cell

To assess the dynamics of each blood component over time, transfusion data were grouped by year and month to identify trends throughout the study period. Although both departments displayed similar overall patterns in blood component usage, irradiated PC emerged as the most frequently requested plasma component, particularly in the Inpatient Department. In contrast, RBC components (specifically washed, filtered, filtered and washed, and irradiated and washed) were more commonly used in the Outpatient Department. The distribution and dynamics of each blood component by department are illustrated in Figure 1.

The transfusion patterns differed significantly between departments over the years. Fisher’s Exact Test revealed statistically significant differences in annual transfusion frequencies between departments for multiple components (Figure S1)

The area under the curve (AUC) for each component across departments is presented in Figure S2. Although many components showed similar AUC values between departments, RBC components (except phenotyped & irradiated RBCs), as well as FFP and certain PC subproducts, exhibited higher AUCs in the Outpatient Department.

Monthly distribution patterns varied considerably; while some components showed peaks during specific periods, others followed different trajectories. These differences in demand between departments are likely influenced by the clinical profiles and treatment needs of the patients they serve.

### Yearly seasonality on blood transfusion requirement per month of the year

When analyzing monthly dynamics, transfusion patterns for components differed significantly between departments when stratified by month (Figure S3). For most of these components, peak transfusion volumes occurred in the middle of the year, suggesting a potential seasonal influence on demand.

### Logistic regression analysis of daily trends in blood component usage

Given the apparent seasonal variation in component demand, this study investigated the influence of time (measured in days) on transfusion dynamics across the study period. Although daily transfusion numbers fluctuated, overall usage patterns between departments remained relatively consistent.

This study utilized a Poisson logistic regression model to analyze how the demand for various blood components changed over time (measured in days). While daily numbers fluctuated, the analysis identified clear, statistically significant temporal trends for nearly every component studied.

The majority of blood components, including FFP, PC, and various RBC products, showed a slight but statistically significant decrease in usage per day.

Conversely, three specific components (Cryo, Irradiated PCa, and Filtered & Irradiated RBCs) showed an increasing trend in demand over the study period (Table 2).

**Table 2:** Statistically significant daily trends in component usage via poisson logistic regression (p-value ≤0.001).

Blood Component	Daily Percentage Change
PCa (irradiated)	+0.19
RBC (filtered & irradiated)	+0.10
Cryo	+0.03
PC (irradiated)	−0.007
FFP	−0.04
RBC (washed)	−0.04
RBC	−0.05
RBC (filtered)	−0.05
RBC (irradiated)	−0.09
RBC (phenotyped)	−0.10
PC	−0.16
PCa	−0.20
RBC (phenotyped & filtered)	−0.26
PC (filtered & irradiated)	−0.50

FFP: Fresh Frozen Plasma; PC: Platelet Concentrate; PCa: Platelet Concentrate by apheresis; RBC: Red Blood Cells; Cryo: Cryoprecipitate.

The Outpatient Department demonstrated a distinct shift in transfusion requirements compared to inpatient services. There was a significant decrease in the use of irradiated PCa, irradiated PC, and Cryo.

However, there was a substantial and statistically significant increase in the usage of most other products, particularly RBC variations. Notably, washed RBC, filtered RBC, and combinations of those treatments saw increases exceeding 200% (Table 3).

**Table 3:** The following breakdown compares the usage of blood products in the Outpatient Department relative to the Inpatient Department (the reference group), adjusted for date as a potential confounder.

Blood Product	Percentage Change	p-value
RBC (washed)	+227	<0.001
RBC (filtered)	+205	<0.001
RBC (filtered & washed)	+204	<0.001
RBC	+181	<0.001
RBC (phenotyped & filtered)	+164	<0.001
PC (filtered & irradiated)	+122	<0.001
RBC (washed & irradiated)	+115	<0.001
RBC (phenotyped)	+104	<0.001
PC	+68.7	<0.001
Cryo	−18.3	0.01
PC (irradiated)	−46.6	<0.001
PCa (irradiated)	−61.3	<0.001

**Fig. 2:**
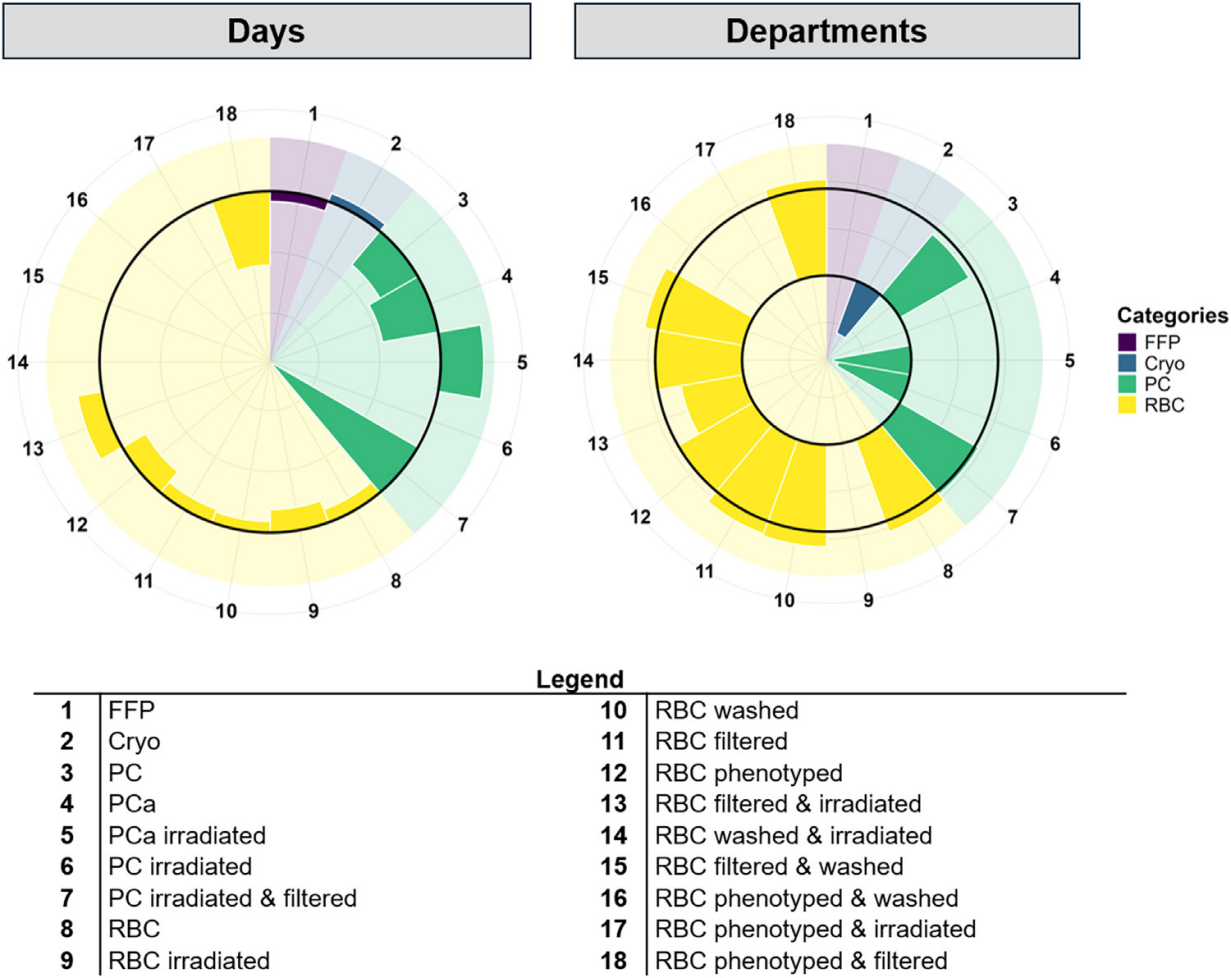
Temporal and interdepartmental dynamics of blood component usage. Left (Days): Poisson regression model illustrating daily percentage changes in component demand. The radial axis (log scale: −0.7 to 0.22) depicts the daily dynamic change; the solid black line represents the baseline (zero change). Usage peaked mid-year, followed by a longitudinal decrease in almost all blood components across the study period. Right (Departments): Comparative usage between departments, with the Inpatient Department serving as the reference group. The radial axis (log scale: −4.5 to 7.0) indicates the percentage change for the Outpatient Department. Concentric black lines represent the baseline (inner circle, value of 0) and the 100% increase threshold (outer circle, log value of 100). RBC: Red Blood Cells; PC: Platelet Concentrate; PCa: Platelet Concentrate by apheresis; FFP: Fresh Frozen Plasma; Cryo: Cryoprecipitate

RBC: Red Blood Cells; PC: Platelet Concentrate; PCa: Platelet Concentrate by apheresis; Cryo: Cryoprecipitate.(Figure 2).

### Data-driven forecasting of blood product usage: strategies for hospital resource planning

To estimate the future demand for blood components, the focus was on the components that showed an increasing trend over time based on the day-day analysis, specifically Cryo, irradiated PCa, and filtered & irradiated RBC. A predictive ARIMA model was trained using data from January 2019 to December 2022 to forecast usage patterns for 2023, with model performance validated by comparing predicted values against actual data. An automatic model selection process was used to identify the optimal ARIMA configuration for each component, with selection based on minimal residuals as well as an evaluation of Autocorrelation Function and Partial Autocorrelation Function diagnostics (Figure S4).

**Fig. 3:**
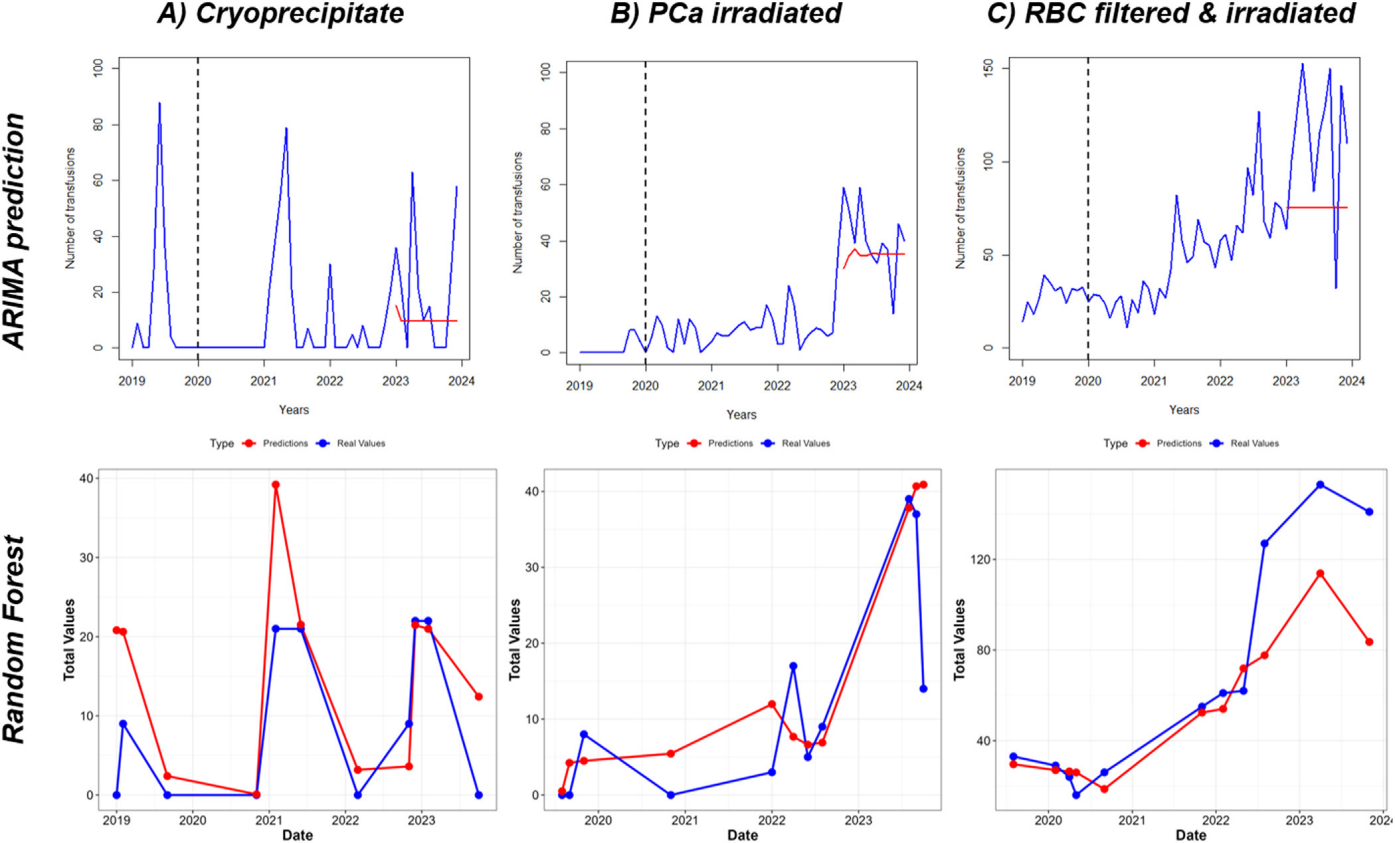
Prediction of blood components for 2023 based on auto regressive integrated moving average (ARIMA) model. The prediction of blood components by the ARIMA model and Random Forest analysis regarding the usage of increased blood components: (A) cryoprecipitate, (B) irradiated Platelet Concentrate by apheresis (PCa) and (C) filtered & irradiated Red Blood Cells (RBC). Red lines represent the predicted values in the models, while blue lines are the real values observed. The dashed line represents the beginning of 2020, when the COVID-19 pandemic started.

Based on the training data, the ARIMA model predicted an initial increase in 2023 followed by stabilization for both Cryo and irradiated PCa. The usage of filtered and irradiated RBCs was forecasted to remain stable throughout the year. However, actual 2023 data revealed more frequent and pronounced peaks in Cryo usage compared to previous years. The model demonstrated a strong fit (RMSE = 17.81), and the Ljung-Box test confirmed the absence of significant autocorrelation in the residuals (χ^2^ = 32.9; p-value = 0.1), indicating a robust model for the underlying trend. Similarly, although irradiated PCa followed an upward historical trend and the model showed a good fit (RMSE = 9; Box-Ljung test χ² = 14.2; p-value = 0.94), its variability in 2023 exceeded that observed in the training period. For Filtered and irradiated RBC, while the model predicted a stable trend (RMSE = 21.7; Box-Ljung test χ² = 16.2; p-value = 0.87), actual usage in 2023 included a peak higher than any seen during the training period (Figure 3). Overall, the predicted values were consistently lower than the values observed in 2023. Nonetheless, the model captured the temporal structure of the data well, as indicated by the non-significant results of the Box-Ljung tests. This discrepancy between predicted and actual values highlights both the increasing demand for these blood components and the challenges involved in modeling such fluctuations. These findings reinforce the importance of robust forecasting tools to support healthcare planning and resource allocation.

Additionally, a random forest model was applied using the same dataset, this time considering all usage patterns collectively. In several instances, the predicted values from the random forest model were higher than the observed values, suggesting that it may be a more robust tool for capturing complex patterns in component usage. This approach could enhance predictive capacity and support the development of more responsive and efficient blood management and hospital policy strategies (Figure 3).

### Transfusion reaction profile across departments

Throughout the study period, the number of reported transfusion reactions was monitored. A reduction was observed in both departments from the beginning of the analysis; however, an upward trend began in 2022. While the Outpatient Department showed a slight increase, the Inpatient Department experienced a more pronounced rise in transfusion reactions (Figure S5). Due to data limitations, it was not possible to analyze the reactions by specific blood component, thus, only the overall incidence across departments could be assessed.

## Discussion

The current study reflects the transfusion reality of a reference center for hemotherapy procedures located in the northern region of Brazil. The patient profile is predominantly onco-hematological disorders. A temporal analysis of transfusion dynamics of two specialized transfusion therapy departments can contribute to strategies and local patient blood management. Interestingly, this study identified consistent increases in Cryo, irradiated CPa, and filtered & irradiated RBC over a five-year period.

The higher utilization of irradiated components observed in the present study may be attributed to the presence of an active transfusion committee. This committee establishes measures to enhance transfusion safety, minimize the risk of transfusion-associated graft-versus-host disease, and reduce dispensing errors in critically ill onco-hematological patients [13,14].

The feasibility of predicting future blood component usage was demonstrated using a Random Forest modeling approach. The model showed strong agreement with the observed values, highlighting its potential for optimizing quality control, improving the monitoring of transfusion reactions, and guiding resource allocation and strategic planning in hospital management. Additionally, the findings emphasize the importance of seasonality in transfusion patterns, which can assist blood donation campaigns, donor screening, and overall blood surveillance.

It is important to acknowledge that the study period overlapped with the COVID-19 pandemic. While studies have reported significant disruptions in blood donation and transfusion patterns during this period, mainly due to social isolation and decreased donor availability [15,16], the data of this study suggest that the trends observed in 2019 were largely maintained. An overall increase in transfusion rates was described previously [12], however, as an effect of the pandemic, a general reduction in certain blood components was noted, particularly RBC products, possibly influenced by a drop in elective surgical procedures and broader shifts in healthcare utilization during the pandemic [15]. The transfusion profile of patients seemed not to change the tendency that was observed in the previous year [2019]. The reduction seen in components, on the other hand, could have occurred either due to natural reductions or due to COVID-19, especially in respect to RBC products.

The mid-year surge in transfusion demand aligns with seasonal trends observed in other regions, especially during holidays and school vacations, when accidents and emergency procedures tend to rise [17]. Interestingly, this pattern differs from other Brazilian hospitals, such as those in Minas Gerais, where distinct temporal trends have been reported [18]. Notably, seasonal factors also affect donor availability, as holidays and family vacations coincide with a reduction in available donors [19]. In Brazil, summer school holidays occur from late December to February, with another break in July. These periods involve family travel and even affect physician availability, which may contribute to the different healthcare service patterns observed later in the year [17].

Higher demands for Cryo, irradiated PCa, and irradiated PC products were observed in the Inpatient Department, which handles acute and emergency cases. These components are often used in patients experiencing coagulopathies, undergoing oncological treatment or bone marrow transplants, or requiring hemostatic support for surgical procedures [20, 21, 22, 23], all of which are the profile of patients attended by this department. The use of irradiated products is particularly relevant for onco-hematological patients to prevent transfusion-associated graft-versus-host disease [24, 25, 26]. Given that this hospital is the state reference center for hematological disorders [27], it is expected that such specialized components would be more frequently used in this setting.

The profile of patients in the Inpatient Department, who often present with acute bleeding conditions, supports the higher usage of hemostatic components. Despite the limited literature on blood component usage across hospital departments, the findings of this study underscore the need to tailor transfusion strategies to patient profiles and departmental needs [24]. Additionally, the increasing incidence of leukemia in the state of Amazonas [28] may reflect improved diagnostic capabilities and greater access to treatment, contributing to the rising demand for components like Cryo and irradiated PCa, which are recommended to minimize transfusion-related adverse events [29, 30, 31]. Given that patients treated at this institution often require frequent transfusions, an observed increase in certain components is expected. However, hospitals dealing with broader patient populations may exhibit different transfusion profiles.

The application of machine learning to healthcare data management, particularly in transfusion medicine, remains limited. While we utilized logistic regression, ARIMA and random forest models, there is potential for broader implementation of predictive analytics. To our knowledge, the study by Mitterecker et al. [32] remains the only work applying machine learning techniques to predict transfusion practices in general patient populations. In contrast, the present study focuses on a longitudinal assessment of a highly transfusion-dependent cohort, emphasizing the need for future research incorporating patient characteristics and follow-up data to refine transfusion management strategies.

An increase in reported transfusion reactions beginning in 2022 likely reflects the impact of a targeted training initiative introduced that year. This program included clinical case discussions, multidisciplinary education sessions, and regular departmental meetings. Consequently, reactions that previously went unrecognized began to be correctly identified and reported. This phenomenon is well-documented in Brazil and in other countries, where underreporting is often due to limited technology and data management infrastructure [33]. Sobral et al. [34] reported that 26% of RBC transfusions in the Federal District had no associated reaction report, emphasizing an urgent need for improved hemotherapy safety and documentation systems [18,35].

Time-series analysis remains underutilized in hemotherapy worldwide. Combined with the lack of technological infrastructure to process health data, especially in remote regions, this highlights a critical need for robust blood surveillance systems [33]. The current study contributes to filling this gap by presenting five years of transfusion data from a major blood center in Brazil. Nonetheless, we acknowledge limitations, including potential underreporting and the absence of an electronic system for managing transfusion and adverse reaction data.

A limitation of this study is the lack of data regarding the full transfusion supply chain; our analysis focuses on clinical administration and does not account for prior stages such as donation, processing, storage, or distribution. However, this study primarily examined the infusion phase, which represents a critical point in transfusion practice with direct implications on patient outcomes. Our results provide a better comprehension of blood component patterns from a specialized center, to mitigate further storage protocols and implement new public health strategies and professional training.

The findings of this study demonstrate that the patient’s profile has a direct influence on transfusion demand but more importantly highlights the importance of local analysis to comprehend blood demand heterogeneity in Brazil. Future studies must integrate data from all the steps of the blood transfusion cycle to provide a broader point of view and enhance a national overview of blood usage in the country.

## Conclusions

In recent years, many hospitals have implemented new technologies and tools aimed at identifying innovative administrative strategies with greater efficiency, guiding future public health initiatives with higher impact and improved cost-effectiveness for health systems. The application of machine learning techniques holds considerable promise for enhancing data analysis, interpretation, and decision-making across various healthcare environments, including hospital settings. However, the application of such approaches within the field of transfusion medicine remains limited, due to both intrinsic and extrinsic challenges that hinder the development of effective public health measures in this domain.

The present study identified a consistent increase in the use of irradiated platelet concentrates (irradiated PC and irradiated PCa) and Cryo over the past five years, reflecting a growing demand for these components among patients with hematological and onco-hematological conditions. Furthermore, stratification by hospital department revealed that the Inpatient Department, responsible for urgent care, exhibited a higher demand for blood components associated with hemostatic support, consistent with the underlying disease burden in this particular patient population.

The data presented in this study contribute not only to local hospital planning but also to a broader understanding of transfusion practices. Analyzing temporal trends and integrating data through advanced technological tools should be considered a priority. These processes are vital for optimizing clinical decisions and guiding management strategies in transfusion medicine.

## Author contributions

Conceptualization: T.A.L., J.S.C., A.L.S.-J. and E.C.C.; Data curation: C.H.A.C., T.R.A., A.L.S.-J. and E.C.C.; Formal analysis: J.A.C.C. and A.L.S.-J.; Funding acquisition: A.L.S.-J. and E.C.C.; Investigation: C.H.A.C., T.R.A. and J.A.C.C.; Methodology: C.H.A.C., T.R.A. and J.A.C.C.; Project administration: T.A.L., J.S.C., A.L.S.-J. and E.C.C.; Supervision: J.S.C., A.L.S.-J. and E.C.C.; Visualization: J.A.C.C. and A.L.S.-J.; Writing – original draft: C.H.A.C., T.R.A., and J.A.C.C.; Writing – review & editing: T.A.L., J.S.C., A.L.S.-J. and E.C.C. All authors reviewed and approved the final draft of the manuscript.

## Ethics approval statement

Prior to data collection, this study was approved by the ethics committee of the Fundação Hospitalar de Hematologia e Hemoterapia do Amazonas (HEMOAM) under protocol number #6.575.757.

## Funding information

C.H.A.C. and T.R.A. holds an undergrad scholarship from Fundação de Amparo à Pesquisa do Amazonas (FAPEAM) via Programa de Apoio à Iniciação Científica from HEMOAM (PAIC—HEMOAM) for this work. J.A.C.C. and E.C.C. hold a MSc and PhD scholarship, respectively, from Fundação Coordenação de Aperfeiçoamento de Pessoal de Nível Superior (CAPES). A.L.S.-J. holds a post-doctoral fellowship from Fundação de Amparo à Pesquisa do Estado de São Paulo (FAPESP). Any of the funders had any decision on conceptualization, design, data collection, analysis, decision to publish nor preparation of this manuscript.

## Data availability

The data that support the findings of this study are available from the corresponding author upon reasonable request.

## Conflicts of interest

The authors declare no conflicts of interest.

## Appendix Supplementary materials

Fig. S1 Usage of each component comparing between both departments by Fisher Exact Test comparison. The number of transfusions was summed by year and expressed in the graphs, RBC: Red Blood Cells; PC: Platelet Concentrate; PCa: Platelet Concentrate by apheresis; FFP: Fresh Frozen Plasma; Cryo: Cryoprecipitate Image, image 1 dummy alt text

Fig. S2 AUC description values of each blood component curve throughout the years observed. H: Hospitalization; T: Transfusion, RBC: Red Blood Cells; PC: Platelet Concentrate; PCa: Platelet Concentrate by apheresis; FFP: Fresh Frozen Plasma; Cryo: Cryoprecipitate Image, image 2 dummy alt text

Fig. S3 Blood use per month comparing departments, **p* < 0.05; ***p* < 0.01; ****p* < 0.001; ns: not significant, Cryo: Cryioprecipitate; FFP: Fresh Frozen Plasma; PC: Platelet Concentrates; PCa: Platelet Concentrates by apheresis; RBC: Red Blood Cells Image, image 3 dummy alt text

Fig. S4 Residuals, histograms, Autocorrelation Function and Partial Autocorrelation Function of each blood component with white noise in ARIMA model, Residual production on data regarding A) Cryoprecipitate (AIC = 522.16; BIC = 528.44), B) irradiated PCa (AIC = 434.2; BIC = 440.43) and C) Filtered & irradiated RBC (AIC = 538.7; BIC = 544.92) with ARIMA model and best result obtained using an automatic function. RBC: Red Blood Cells; PCa: Platelet Concentrate by apheresis Image, image 4 dummy alt text

Fig. S5 Frequency of transfusion reactions by year and department, Note: Data represent reported occurrences; specific etiologies of reactions were not available for this analysis. Image, image 5 dummy alt text

## Footnotes

Supplementary material associated with this article can be found in the online version at doi:10.1016/j.htct.2026.106460.
