# Supplementary figures and images for: Hemovigilance in a Brazilian Amazon blood center: A temporal analysis of 5-year consumption patterns
Source: Hematol Transfus Cell Ther. 2026 May 13;48(3):106460. doi: 10.1016/j.htct.2026.106460 (PMC13196408; doi:10.1016/j.htct.2026.106460)

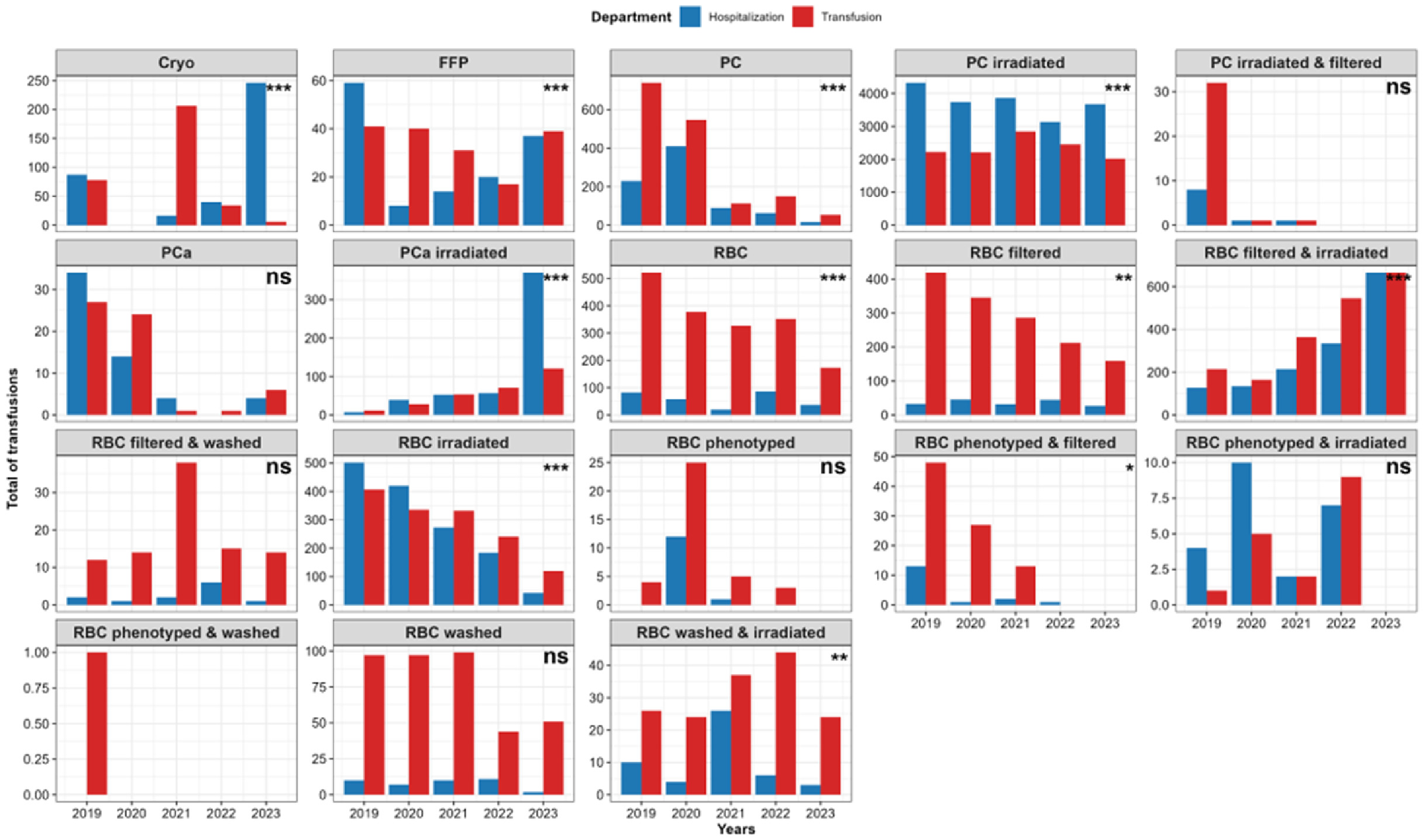

Supplement: Supplementary file 1 — Fig. S1 Usage of each component comparing between both departments by Fisher Exact Test comparison. The number of transfusions was summed by year and expressed in the graphs, RBC: Red Blood Cells; PC: Platelet Concentrate; PCa: Platelet Concentrate by apheresis; FFP: Fresh Frozen Plasma; Cryo: Cryoprecipitate [file mmc1.jpg]

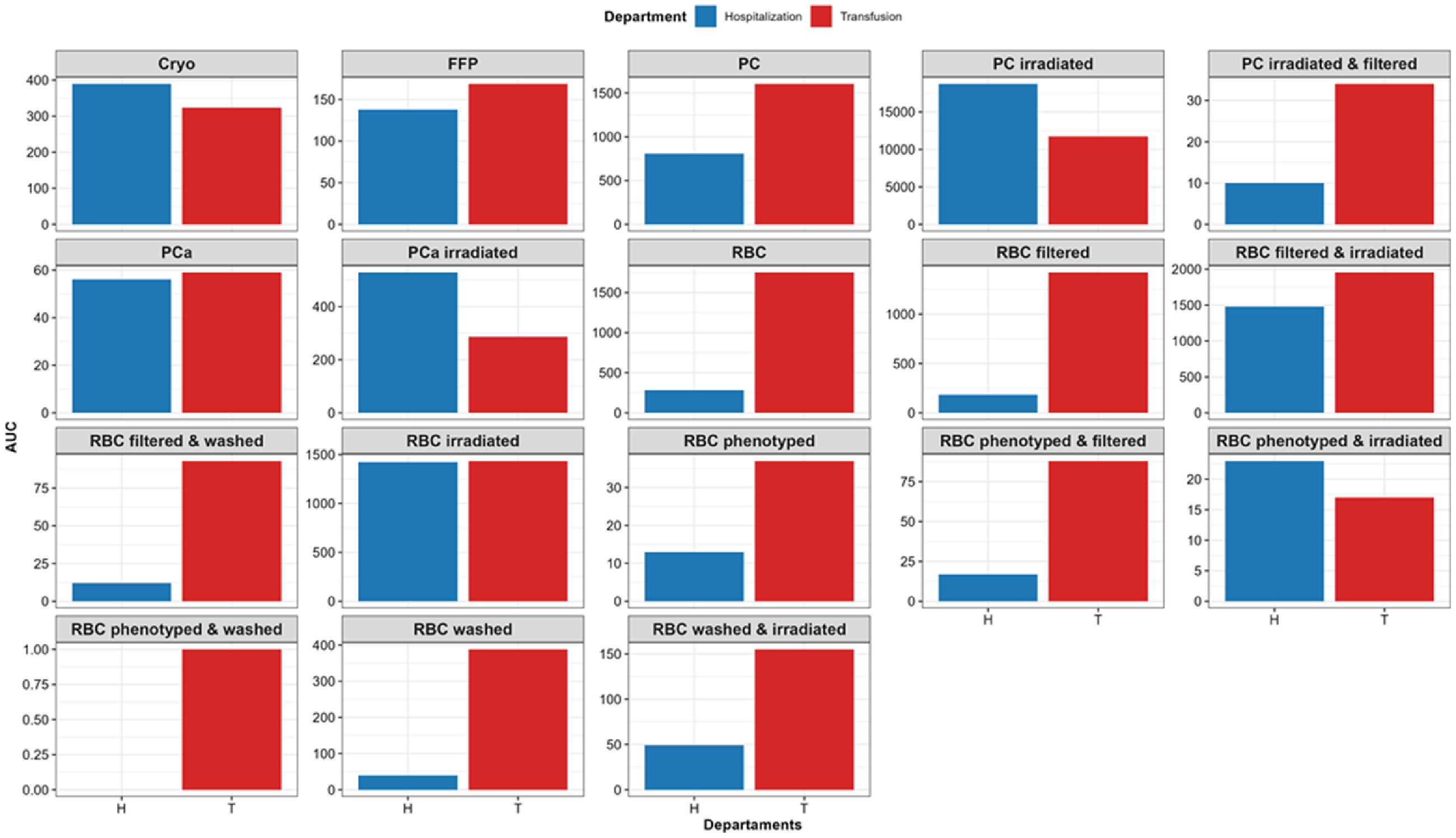

Supplement: Supplementary file 2 — Fig. S2 AUC description values of each blood component curve throughout the years observed. H: Hospitalization; T: Transfusion, RBC: Red Blood Cells; PC: Platelet Concentrate; PCa: Platelet Concentrate by apheresis; FFP: Fresh Frozen Plasma; Cryo: Cryoprecipitate [file mmc2.jpg]

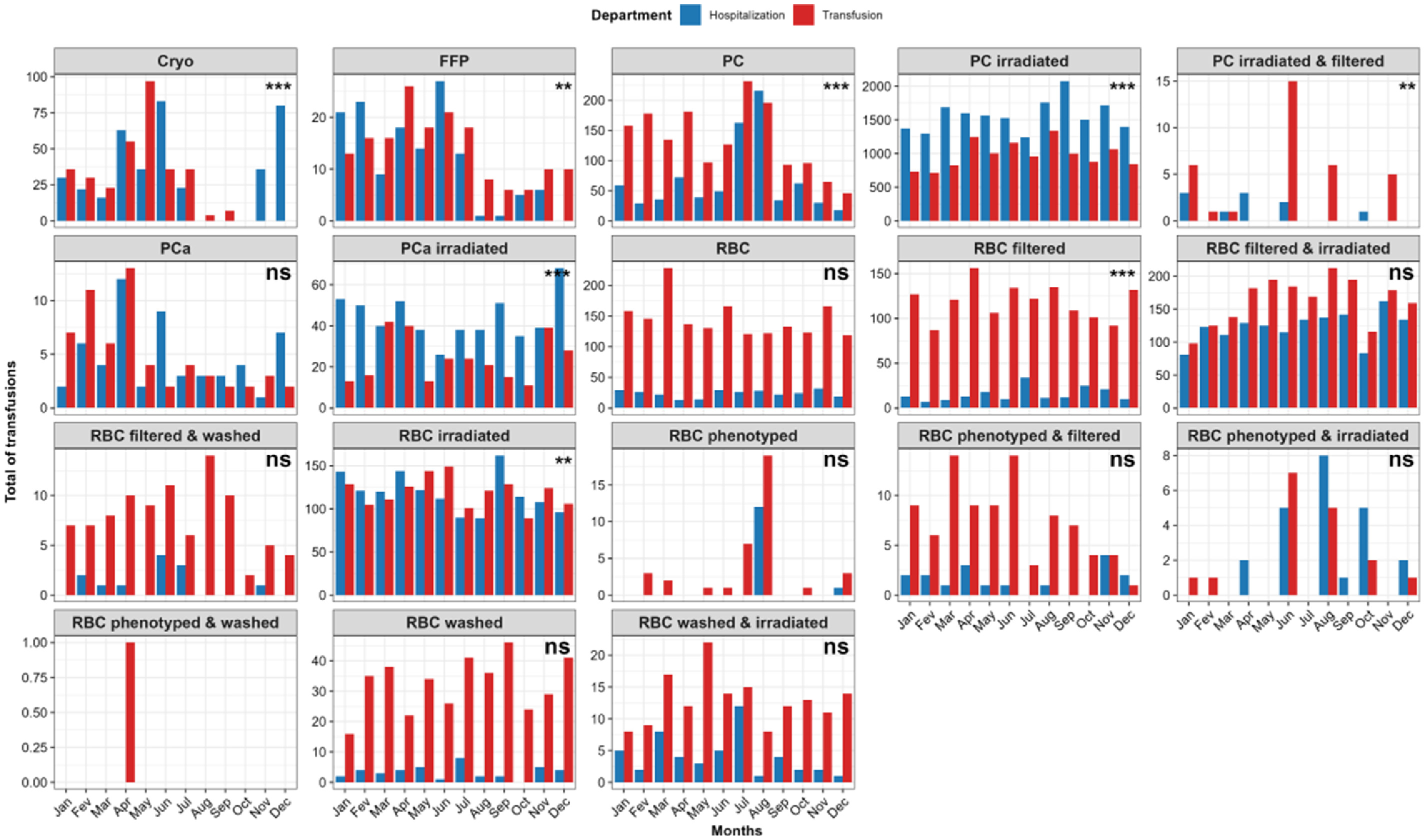

Supplement: Supplementary file 3 — Fig. S3 Blood use per month comparing departments, *p < 0.05; **p < 0.01; ***p < 0.001; ns: not significant, Cryo: Cryioprecipitate; FFP: Fresh Frozen Plasma; PC: Platelet Concentrates; PCa: Platelet Concentrates by apheresis; RBC: Red Blood Cells [file mmc3.jpg]

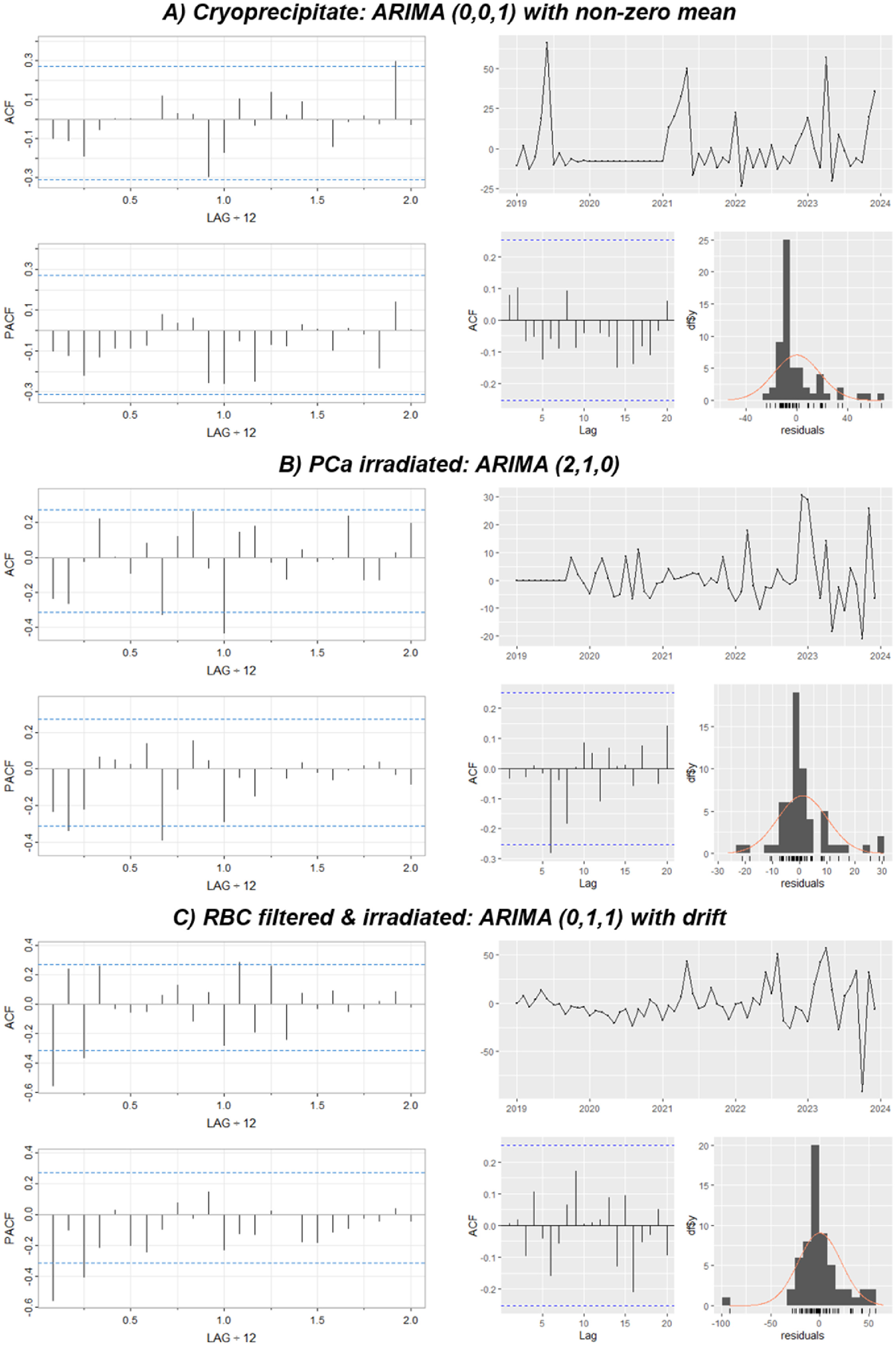

Supplement: Supplementary file 4 — Fig. S4 Residuals, histograms, Autocorrelation Function and Partial Autocorrelation Function of each blood component with white noise in ARIMA model, Residual production on data regarding A) Cryoprecipitate (AIC = 522.16; BIC = 528.44), B) irradiated PCa (AIC = 434.2; BIC = 440.43) and C) Filtered & irradiated RBC (AIC = 538.7; BIC = 544.92) with ARIMA model and best result obtained using an automatic function. RBC: Red Blood Cells; PCa: Platelet Concentrate by apheresis [file mmc4.jpg]

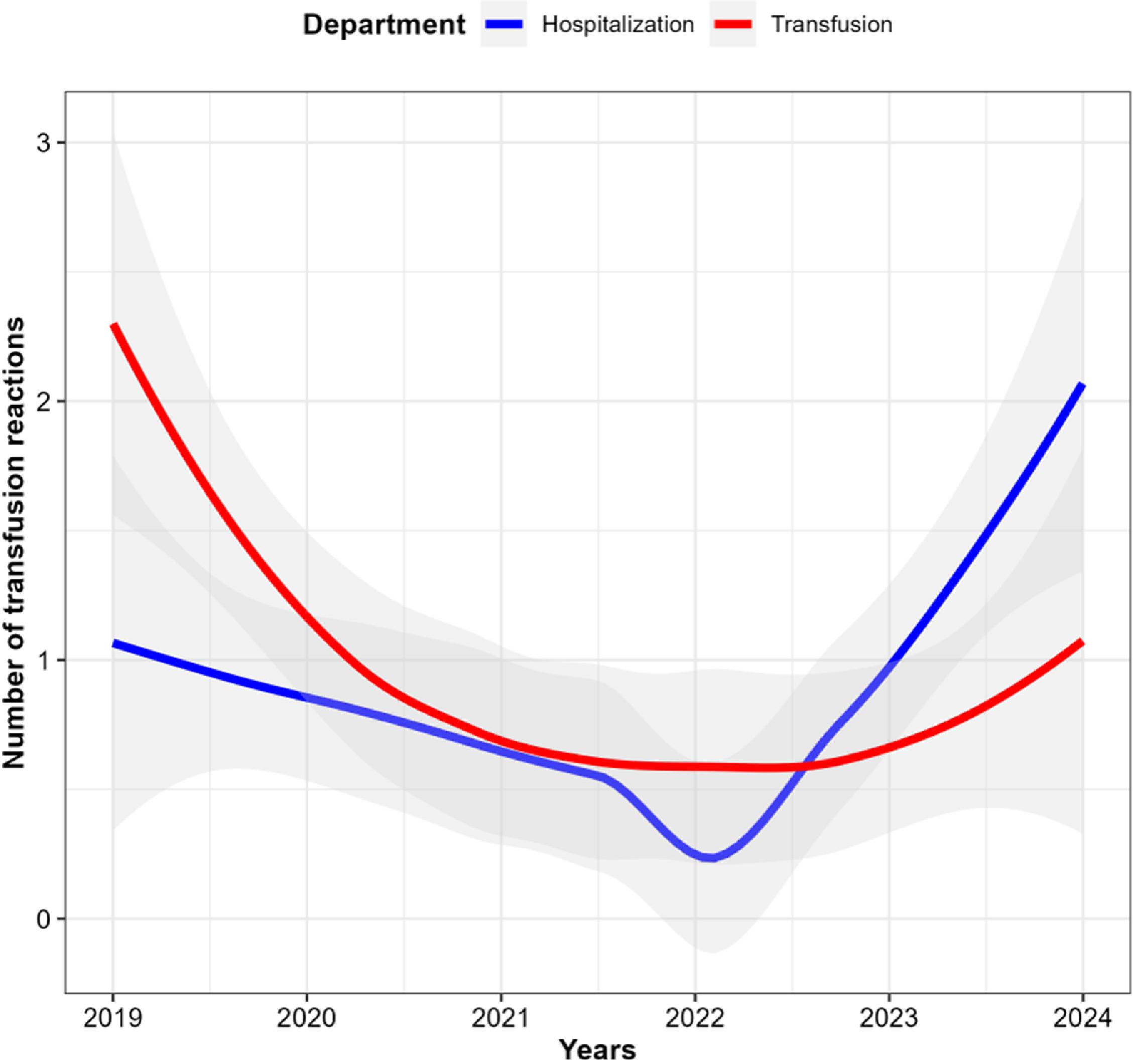

Supplement: Supplementary file 5 — Fig. S5 Frequency of transfusion reactions by year and department, Note: Data represent reported occurrences; specific etiologies of reactions were not available for this analysis. [file mmc5.jpg]
